# Isolation and characterization of a temperature-sensitive lethal strain of *Anopheles arabiensis* for SIT-based application

**DOI:** 10.1186/s13071-018-3216-7

**Published:** 2018-12-24

**Authors:** Cyrille Ndo, Yacouba Poumachu, Danale Metitsi, Herman Parfait Awono-Ambene, Timoléon Tchuinkam, Jeremie Lionnel Roger Gilles, Kostas Bourtzis

**Affiliations:** 1 Institut de Recherche de Yaoundé (IRY), Organisation de Coordination pour la lutte contre les Endémies en Afrique Centrale (OCEAC), P.O Box 288, Yaoundé, Cameroon; 2 Faculty of Medicine and Pharmaceutical Sciences, University of Douala, Douala, Cameroon; 3 Centre for Research in Infectious Diseases (CRID), P.O Box 13591, Yaoundé, Cameroon; 4 Vector Borne Parasitic and Infectious diseases Unit of the Laboratory of Applied Biology and Ecology (VBID-LABEA), Departement of Animal Biology, Faculty of Sciences of the University of Dschang, P.O Box 067, Dschang, Cameroon; 5 Faculty of Sciences, University of Yaoundé I, P.O. Box 337, Yaoundé, Cameroon; 6 Insect Pest Control Laboratory, Joint FAO/IAEA Programme of Nuclear Technique in Food and Agriculture, Vienna, Austria

**Keywords:** *Anopheles arabiensis*, sterile insect technique, genetic sexing strain, *Plasmodium*, malaria, ethyl methylsulfonate, temperature-sensitive lethal strain

## Abstract

**Background:**

Malaria is still a global health problem and vector control is the cornerstone of disease control strategies using indoor residual insecticide spraying (IRS) and insecticide-treated nets. The situation *is becoming* acute with widespread resistance to the limited arsenal of available *insecticide* classes. Therefore, new and innovative tools to reduce *Plasmodium* transmission are in need and this situation raised considerable interest in using sterile insect technique (SIT) against human pest insects, particularly *Anopheles* malaria vectors. When considering a mosquito release programme, one of the first issues to be addressed is how to eliminate/separate the hematophagous vector females. In this paper, we report the development and evaluation of an *Anopheles arabiensis* temperature-sensitive lethal (tsl) strain towards its potential use for the development of a genetic sexing strain (GSS).

*Anopheles arabiensis* male mosquitoes originated from North Cameroon were treated with 0.05% ethyl methanesulfonate (EMS). The mutagen was added to 10% sucrose solution and mosquitoes fed *ad-libitum* for 24h and 48h on a cotton wool soaked with sucrose-mutagen solution placed at the top of the cup. Treated males were then allowed to mate with wild virgin females and their progenies were screened for heat sensitivity from F3 to F8, until the isolation and establishment of a tsl strain which was further characterized by assessing its productivity (fecundity and fertility), larval development, adult longevity as well as nature and inheritance pattern of the tsl.

**Results:**

Observations showed that the number of eggs laid and their hatch rate were similar between females that mated with mutagenized males and those from the control suggesting that mutagenesis didn't affect *An. arabiensis* male fecundity and fertility. At F3, fourteen isofemale families out of 88 tested, which showed mortalities ranged between 50 and 80 %, were selected as lines potentially containing a tsl mutation. From F4 to F8, a tsl strain was isolated and established by screening L1 larvae at 41°C for 3 hours. This strain showed similar life history traits compared to the wild type strain in terms of fertility, larval development time and adult's emergence. Crossing experiments to further assess the nature and inheritance pattern of the tsl phenotype showed that it is due to a recessive allele located on an autosome.

**Conclusion:**

The successful establishment of the *An. arabiensis tsl* strain is a valuable tool towards the development of a GSS for SIT applications against this species. This will be done by induction of a Y-autosome translocation to link the wild-type allele to the Y chromosome in such manner that males are wild-type (temperature resistant) and females are mutant (temperature sensitive).

## Introduction

Malaria is caused by the *Plasmodium* parasites which are transmitted to humans through the bite of infected female *Anopheles* mosquitoes. Despite efforts made for diagnosis and treatment of the disease and for vector control, transmission still occurs in 96 countries in the tropics of the Americas, many parts of Asia and most of Africa. In 2016, an estimated 216 million cases of malaria occurred worldwide with 445000 deaths. Sub-Saharan Africa was reported to be the most affected by the disease, where an estimated 80% of malaria deaths occurred [1].

In addition to drug administration, the control of malaria mainly relies on the ability to suppress mosquito vector populations or to prevent human-vector contacts using conventional methods such as indoor residual insecticide spraying (IRS) and insecticide-treated nets, respectively [2]. However, the efficacy of these methods is seriously compromised by the emergence and spread of insecticide resistance in mosquitoes, and there are also legitimate environmental and human health concerns about many insecticides used for vector control [3–5]. Henceforth, it is unanimously recognized that current control methods are insufficient to achieve and maintain malaria elimination, pointing out the necessity to develop new and innovative tools to reduce *Plasmodium* transmission, which can integrate with and enhance current malaria control measures [6, 7].

The sterile insect technique (SIT) is among the innovative vector control methods envisaged. It consists of the production and release of large number of sterile males that mate with wild females preventing production of offspring and it is always recommended to be applied as a component of an area-wide integrated pest management strategy [8–13]. SIT has proven to be a safe, cost-effective and environmentally friendly strategy, and has been successfully used to control a number of insect pest species including populations of screwworm fly (*Cochliomyia hominivorax*), Mediterranean fruit fly (*Ceratitis capitata*) and tsetse fly (*Glossina austeni*) [10, 14]. The experience and knowledge gained from implementation of this technique in other insect species has resulted in considerable interest in using SIT to control insect vectors of human diseases, particularly for malaria vector control.

In SIT program targeting malaria vectors, only sterile males should be released since females could transmit the disease as they are blood suckers. Besides, bisexual releases could significantly reduce the efficacy and cost-effectiveness of the application, because released males and females would mate with each other reducing the proportion of mating between sterile males and wild females [15]. Therefore, one of the crucial steps in the development and implementation of a mosquito SIT program is sex separation and female elimination [16]. Sex separation and female elimination must ideally take place as early as possible during the development (eg: eggs or L1) as this minimizes rearing cost and would overall facilitate the handling and processing of male only-based sterile releases. Additionally, the method developed for sex separation and female elimination should ideally be conditionally lethal for females because they are needed to maintain the colony and for male production [16, 17].

Genetic sexing strains for sex separation and female elimination have been developed for *Anopheles* species (*Anopheles albimanus* and *An. arabiensis*) using classical genetic approaches based on the Y-autosome translocation of an insecticide resistance mutation to link the inheritance of this mutation to sex [17]. For example, the males of the *An. arabiensis* ANO IPCL1 genetic sexing strain are resistant to dieldrin, while females are eliminated when exposing eggs or other stages to the insecticide. This method proved to be efficient leading to complete elimination of females, without significant decrease of male emergence, when eggs were exposed to 2, 3, and 4 ppm dieldrin solution [18, 19]. However, there have been several concerns about this strain in respect to its productivity, stability as well as the potential impact on environment [20]. In addition, it has been shown that dieldrin becomes less potent once used, probably because molecules are absorbed by eggs as well as onto the surfaces of containers in which the treatments are performed, preventing multiple uses of a dieldrin solution for consecutive treatments [21]. Consequently, treatments of large number of eggs, as it would be the case for SIT programmes, should produce enormous quantities of waste which need to be managed. Moreover, the fact that chemicals cannot penetrate the eggshell in most of the cases could also limit the efficiency of the chemical-based systems [10]. Therefore, selectable markers that respond to physical treatment (e.g. temperature) could be a better alternative.

In the present study, we report the establishment and evaluation of an *An. arabiensis* tsl strain towards its potential use for the development of a genetic sexing strain, similar to the Mediterranean fruit fly *C. capitata* tsl-based Vienna 8 GSS which is currently used for SIT applications to suppress populations of this major agricultural pest worldwide [22–24].

## Methods

### Anopheles arabiensis mosquito colony

*Anopheles arabiensis* mosquitoes were collected in Mayo-Oulo (9°46'N; 13°44'E), a locality situated within the dry savannah area of North Cameroon. In these sites, the vegetation is constituted of an arid savannah and the climate is characterized by a short rainy season from July to September, with an average annual rainfall of 750 mm. Blood-fed resting females were collected using electric aspirators in human dwellings and external shelters. Subsequently, *An. gambiae* (*s.l.*) mosquitoes were sorted from other species using morphological criteria [25, 26]. Larvae were collected in *An. gambiae* (*s.l.*) typical aquatic habitats using dipping method and brought back to the laboratory. They were fed with TetraMin® Baby fish food until emergence of adults. Once emerged, adults were supplied with 10% sucrose solution for three days and subsequently fed with rabbit's blood meal.

Field-collected or laboratory blood-fed females were kept in cages until they were fully gravid and ready to lay eggs. Then, females were individually placed in plastic cups containing approximately 25 ml of water for oviposition. Once having laid eggs, they were killed and genomic DNA was extracted from legs using a standard protocol [27], followed by species identification by PCR carried out using GeneAmp® PCR System 2700 (Applied Biosystems, UK). Because *An. gambiae* M and S forms (now *An. coluzzii* and *An. gambiae*, respectively) [28] and *An. arabiensis* are the only members of the *An. gambiae* complex reported in North Cameroon, we used the PCR diagnostic method of Santolamazza et al. [29] which allows rapid discrimination between the three species. The PCR is based on the amplification of a nearly 200 bp-long Short Interpersed Nuclear Element (SINE 200) locus on chromosome X (locus S200 X 6.1.) using the primers SINE 200X6.1F (5'-TCG CCT TAG ACC TTG GGT TA-3') and SINE 200X6.1R (5'-CGC TTC AAG AAT TCG AGA TAC-3'). The reaction mixture contained 2 μl of 10x PCR buffer (Promega, Madison WI, USA), 1 mM MgCl_2_, 0.2 mM of each dNTP, 0.3 μM of each primer (Eurogentec, Angers, France), 0.35 unit of Taq DNA Polymerase (Promega) and 2 μl of template DNA, in a total volume of 20 μL. Amplification runs were performed under the following conditions: an initial denaturation step at 94°C for 10 min followed by 35 cycles of 30 s at 94°C, 30 s at 54°C, 60 s at 72°C, and a final elongation step of 10 min at 72°C. In 2% agarose gels, the amplified fragment length is 479 bp in *An. coluzzii*, in which the insertion is fixed, and 249 bp in the *An. gambiae* due to the absence of the insertion. Similarly, the insertion is absent in *An. arabiensis* but the PCR product is represented by a 223 bp band due to a 26 bp deletion in the S200 X 6.1. flanking region. Progenies of all mosquitoes identified as *An. arabiensis* were pooled and reared in the insectary. Larvae were reared at 29 ± 1°C and about 70±10% relative humidity and fed with TetraMin. Adults were maintained at 27 ± 1°C and 80-85% relative humidity with a continuous supply of 10% sucrose solution.

### EMS mutagenesis and establishment of an *An. arabiensis* temperature-sensitive strain

**Fig. 1:**
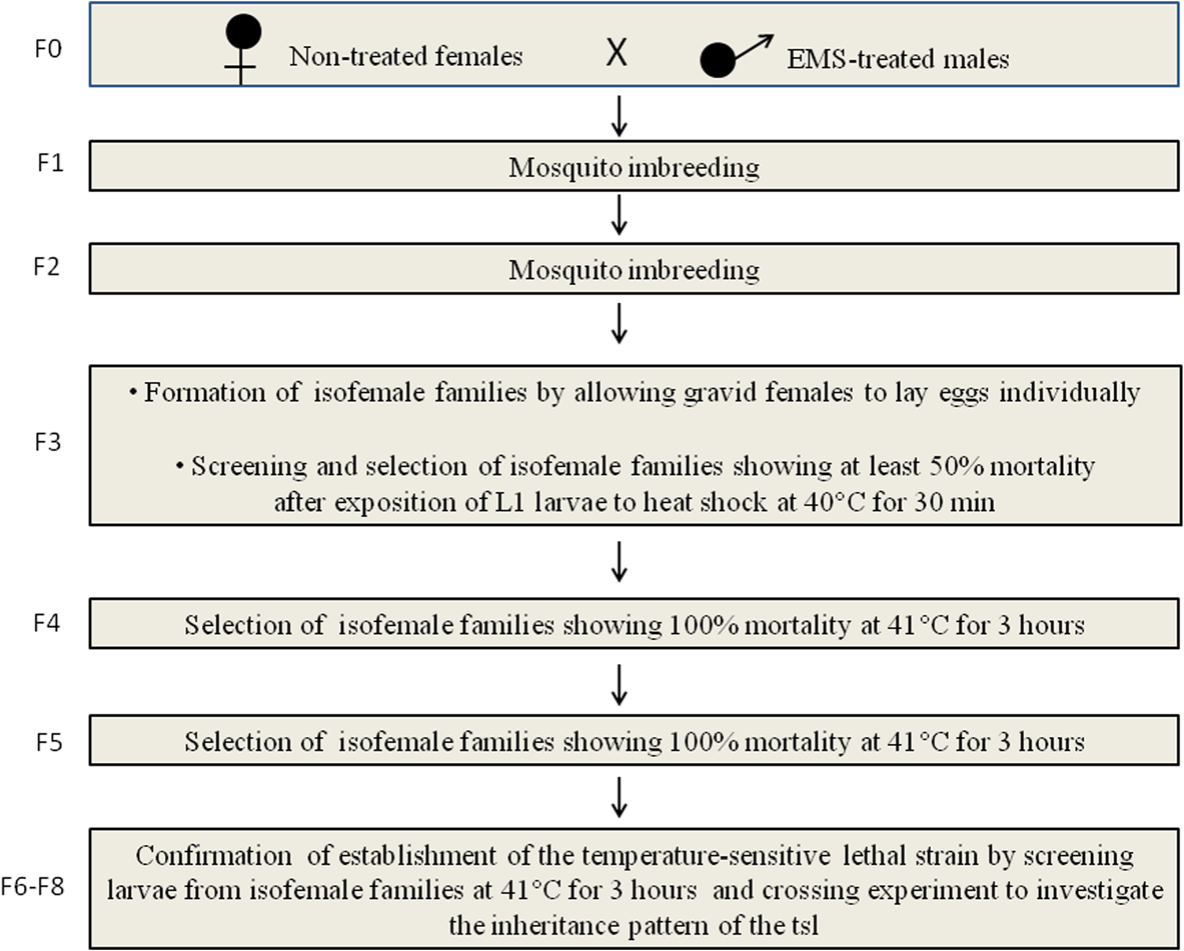
Experimental scheme for the isolation and establishment of an *An. arabiensis* tsl strain

The protocol of selection and establishment of a tsl strain is shown in Fig. 1. Male and female pupae were separated based on the morphology of the last abdominal segment, and they were placed in different cages for emergence of adults. Four-day-old virgin males were treated with 0.05% of EMS obtained from Sigma (Taufkirchen, Germany). This dose has been used to induce temperature-sensitive lethal mutations in *Culex tritaeniorhynchus* using the same mutagen [30]. The mutagen was added to 10% sucrose solution. Three replicates of 50 virgin males per cup were formed and mosquitoes were allowed to feed *ad libitum* for 24 h and 48 h on a cotton wool soaked with sucrose-mutagen solution placed at the top of the cup. After EMS-sugar feeding, EMS-treated males were introduced in cages with virgin females in a 1:1 ratio for 3 consecutive days. After that, females were blood-fed and allowed to oviposit individually. The fecundity (number of eggs laid) and fertility (percentage of eggs hatched) of each female were assessed and larvae pooled according to the treatment for rearing to F3.

Females were blood-fed and allowed to oviposit individually as described above. Eggs were allowed to hatch in source water containing larval diet. L1 larvae were screened for temperature sensitivity as follows. Thirty (*N*=30) L1 larvae (24-30 h after hatching) per isofemale family were introduced in Eppendorf tubes containing 0,5 ml pre-warmed water at 40°C. The tubes containing larvae were placed in a digital bath (Benchmark Scientific Model H2000-4-E) set at 40 °C for 30 min. The remaining larvae were maintained in cups throughout the experiments. Two controls were used: (i) a negative control constituted of larvae from EMS-treated and normal strains which have been handled similarly, but were not exposed to the elevated temperature, to verify that variations in mortality were induced by heat shock; and (ii) a positive control constituted of normal wild type L1 larvae which have been handled similarly and exposed to heat shock at 40°C for 30 min, to verify that increased mortality in EMS-treated strain after heat shock was due to an EMS-induced tsl mutation. Mortalities were recorded 24 h after exposure to the specified temperatures. As we were attempting to isolate a tsl mutation, only families which showed high and significant mortalities compared to the controls were selected as possibly containing a tsl mutation. At F4, the optimal conditions to achieve 100% and lowest mortalities in EMS-treated and control larval bathes, respectively, were identified by screening L1 larvae at various temperatures (25 to 42°C) and time periods (30 min to 120 min). These optimal conditions were used to establish the tsl strain, from F4 to F8.

The nature and inheritance pattern of the tsl was investigated using standard crossing experiments. For each crossing, virgin males and females were placed for four consecutive days in cages in ratio 1:1, and females were blood fed and allowed to oviposit individually. L1 larvae of each crossed strain, and of the tsl and the wild type strains were then screened for temperature sensitivity as described above.

### Characterization of the *An. arabiensis* temperature-sensitive lethal strain

#### Assessing the productivity of the temperature-sensitive lethal strain

Twenty-four hours-old virgin males and females were placed in cages in ratio 1:1 and allowed to mate for three consecutive days. After that, females were blood-fed and gravid females were allowed to oviposit individually. The number of eggs laid by each female was counted under a stereo-microscope. Average values were calculated and compared between the temperature-sensitive and the wild type strains. Eggs were subsequently allowed to hatch in source water and larvae from each batch were counted and removed daily for two consecutive days. Female insemination rate was estimated by dividing the number of females for which eggs hatched by the number which laid eggs. Fertility of each female was calculated by dividing the number of L1 by the number of eggs initially placed in trays for hatching. Similarly, average values were calculated and compared between temperature-sensitive lethal and the wild type strains.

#### Larval development and adult longevity

Mosquito larvae were reared at 25°C and 70% RH. Three replicates of each strain were formed by adding 50 first instars larvae to 0.3 L of source water in 15 × 10 cm plastic pans. Each larva received daily 0.2 mg of food (TetraMine) until pupation. Larval trays were inspected once daily at 10 am, and dead larvae or pupae were removed and recorded. The pupae were transferred to individual cups, and adult emergence and survival were monitored. The following parameters were assessed: larval survival as the proportion of L1 that reached the pupal stage; larval development time as the time between L1 stage and the pupation; pupal survival as the proportion of adults emerged from pupae, and longevity as the number of days adult mosquitoes survived.

#### Data analysis

Data were entered in excel and all statistical analysis was performed using GraphpadPrism software V5.00. Fisher's exact and chi-square tests were used to compare proportions while Mann-Whitney test was used to compare means. The Log-rank (Mantel-Cox) test was used to compare survival curves. All P-values were considered as significant at a cut-off of 0.05.

## Results

### Mosquito species identification

In Mayo-Oulo, a total of 323 *Anopheles* mosquitoes were collected among which 168 (52.01%) were blood-fed, semi-gravid or gravid females at the time of collection. According to morphological identification, *An. gambiae* (*s.l.*) was the most abundant (302/323) and the other species collected in the locality was *An. rufipes* (21/323). In the insectary, only 52 *An. gambiae* (*s.l.*) mosquitoes laid eggs and their molecular identification resulted in the following distribution: *An. gambiae* (43/52), *An. arabiensis* (6/52) and *An. coluzzii* (3/52). All *An. arabiensis* progenies from these six females were used to establish a colony.

### Fecundity and fertility of mutagenized males

Feeding mosquitoes with EMS-sucrose solution for 48h resulted in high sterility. Therefore, experiments were done only with males fed for 24h. Our data showed that fecundity was similar (Mann-Whitney test, *P* = 0.899) between females mated with EMS-treated males (*n* = : 51; range 25–153; mean: 72.22 ± 30; median: 69) and females mated with non-treated males (*n* = 22; range: 10–162; mean: 73.23 ± 40.30; median: 58.50). The hatch rate of eggs laid by females mated with EMS-treated males (mean: 61.13 ± 32.87; median: 75) tended to be lower compared to the control (mean: 74.5 ± 18.56; median: 81), but the difference was not statistically significant (Mann-Whitney test, *P* = 0.282).

### Isolation and establishment of temperature-sensitive lethal strain

**Fig. 2:**
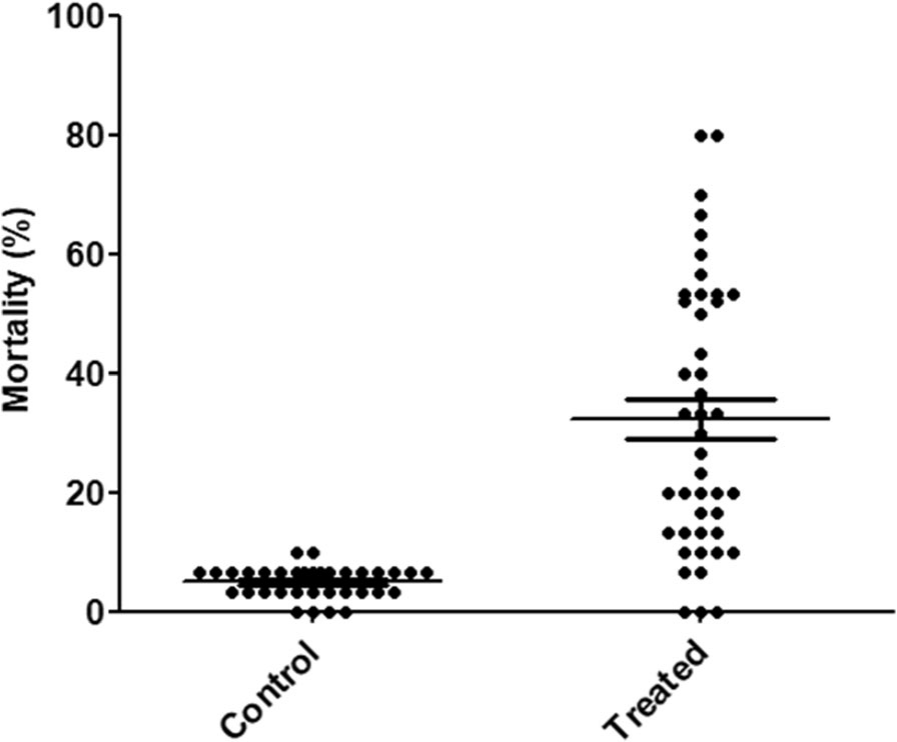
Mortality at F3 in mutagenized (treated, *n* = 2640 larvae from 88 isofemale families) and non-mutagenized (control, *n* = 1800 larvae from 60 isofemale families) mosquitoes after heat shock at 40°C for 30 min. Each dot represents a single isofemale family

At F3, 2640 early first instars larvae originating from 88 isofemale families were screened for their temperature sensitivity after their exposure at 40°C for 30 min. In parallel, 1800 larvae (60 isofemale families) from the control (untreated) were also screened at the same conditions. Mortality was significantly higher (Fisher's exact test, *P* < 0.0001) in the EMS-treated strain (range: 0–80%; mean: 32.36%) compared to the control (range: 0–10%; mean: 5.67%) suggesting that the EMS treatment induced temperature sensitivity in *An. arabiensis* (Fig. 2). Fourteen (15.91%) isofemale families out of 88 tested showed mortality ranging between 50 and 80 % and were selected as possibly containing a tsl mutation.

**Fig. 3:**
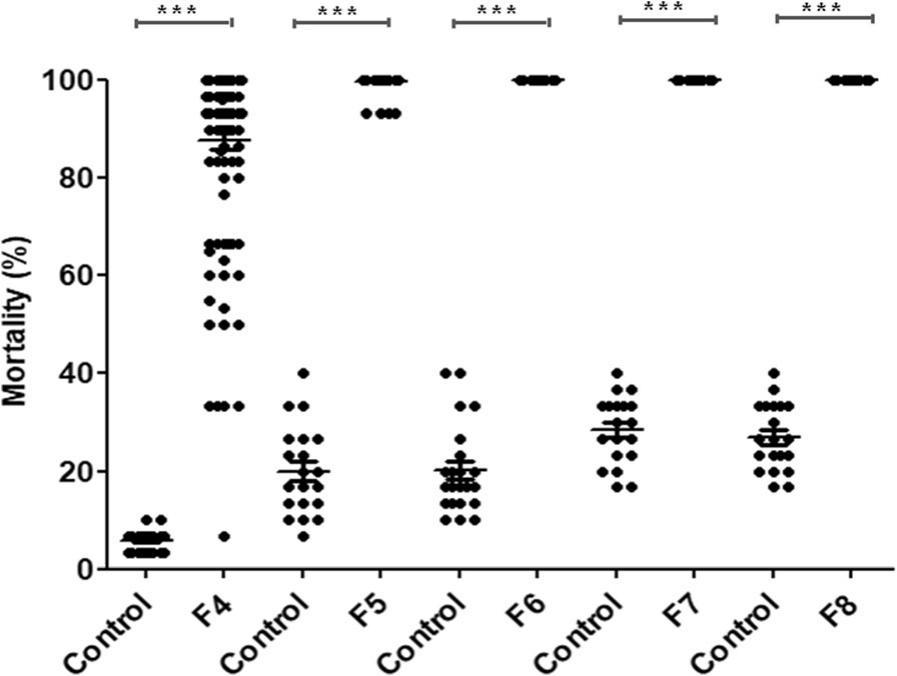
Mortality in mutagenized (F4-F8, *n* = 3480 larvae from 116 isofemale families) and non-mutagenized (control, *n* = 5670 larvae from 189 isofemale families) mosquitoes after heat exposure at 41°C for 3 hours. Each dot represents a single isofemale family. ***P < 0.001

As we were unable to observe 100% mortality after the exposure of L1 mosquitoes at 40°C for 30 min at F3, we tested various temperatures (25 to 42°C) and time periods (30 min to 120 min) in F4 (data not shown). A total of 111 isofemale families (3330 L1 larvae) were screened and 53 of them (47.75%) showed 100% mortality after exposure at 41°C for 3 h. The mosquitoes of these 53 lines were pooled and reared to the next generation. Using the same conditions, 103 isofemale families (95.37%) out of 108 (3240 L1 larvae) screened at F5 showed complete mortality and their larvae were pooled and reared to F6. From F6 to F8, the isolation and establishment of the tsl strain was confirmed by screening 116 isofemale families (3480 L1 larvae): no larva survived after 3 h exposure at 41°C (Fig. 3). In the wild type strain, a total of 189 isofemale families (5670 L1 larvae) were tested during F4 to F8 generation under the same conditions and the mortality was significantly lower (mean: 20–27.69%, *P* < 0.0001) compared to the EMS-treated strain (Fig. 3).

**Fig. 4:**
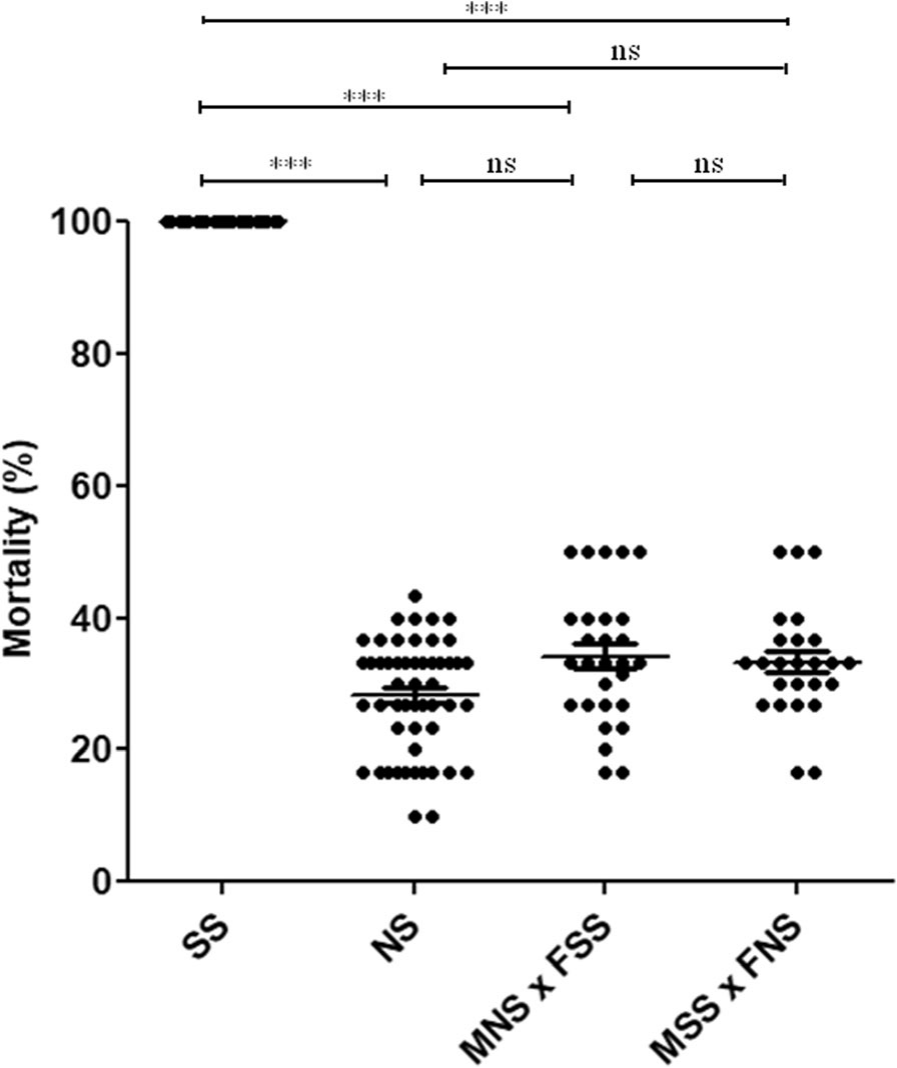
Mortality in temperature sensitive lethal (SS), wild type (NS) and two hybrid strains after heat exposure at 41°C for 3 hours. Each dot represents a single isofemale family. MNS × FSS: males of wild type strain crossed with females of temperature sensitive lethal strain, MSS × FNS: temperature sensitive lethal males crossed with wild type females. A total of 1560 larvae (52 isofemale families) for SS, 1560 (52 isofemale families) for NS, 840 (28 isofemale families) for MNS × FSS and 750 (25 isofemale families) for MSS × FNS were tested. *P < 0.05, **P < 0.01, ***P < 0.001, ns: not statistically different. Comparisons were done two by two

Investigation of the tsl genetic behavior showed that mortality in heterozygous crossed strains had a similar trend compared to that of the wild type strain, although slightly higher, giving preliminary indication that our tsl is recessive and is located on an autosome (Fig. 4).

### Characterization of the temperature-sensitive lethal strain

**Fig. 5:**
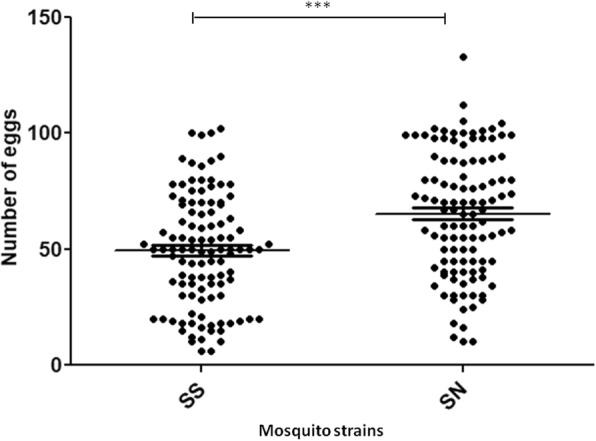
Fecundity of temperature sensitive lethal (SS, *n* = 108 females) and wild type (NS, *n* = 110 females) strains. Each dot represents number of eggs laid by single female. ***P < 0.001

**Fig. 6:**
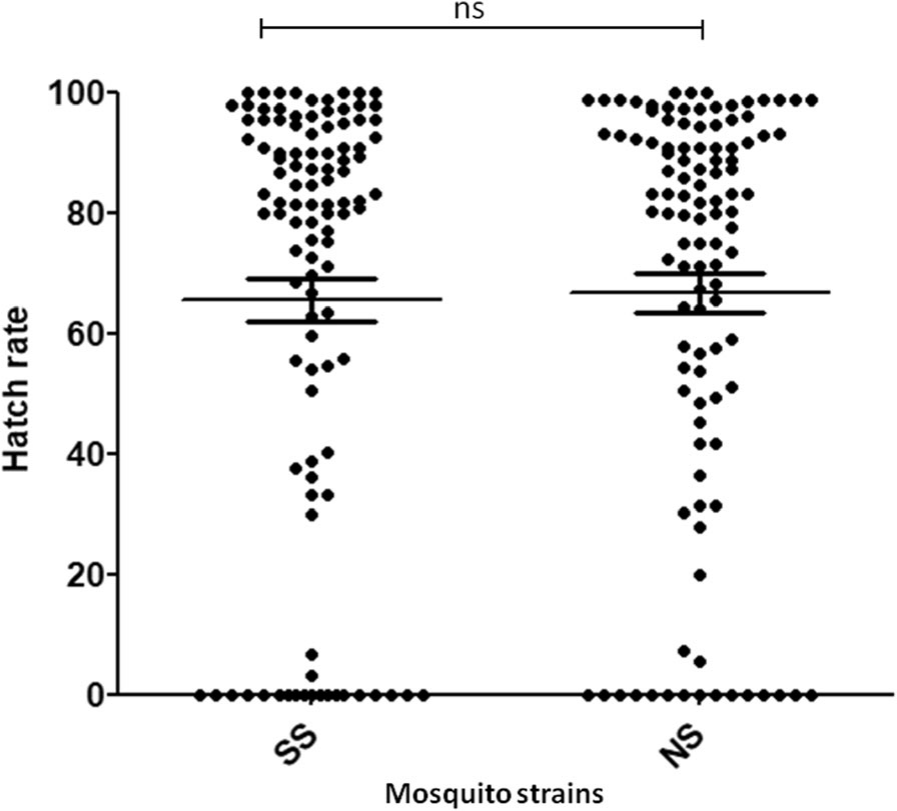
Fertility of eggs laid by females of temperature sensitive lethal (SS) and wild type (NS) strains. Each dot represents hatch rate of eggs from single female. ns: not statistically different

Wild type females tended to lay more eggs than tsl females with the mean number of eggs laid per female being 65.22 ± 26.80 and 49.31 ± 24.16, respectively (Mann-Whitney test, *P* < 0.0001,) (Fig. 5). In contrast to the fecundity, the fertility was similar between the wild type and the tsl strains with the proportion of females for which at least one eggs hatched being 86.36% and 82.41%, respectively (Fisher's exact test, *P* > 0.05). The proportion of eggs that hatched was 69.18% for the wild type strain and 70.54% for the tsl (Chi-square test, *P* = 0.107) (Fig. 6).

**Fig. 7:**
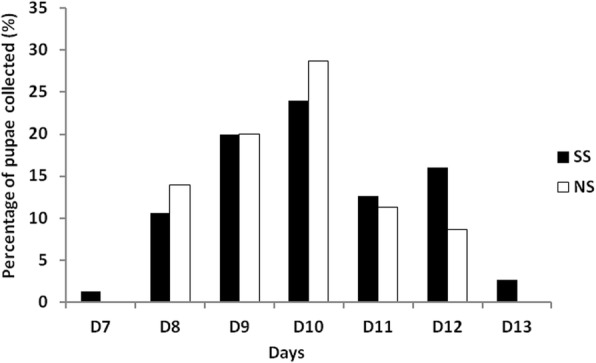
Daily pupation rates of the temperature sensitive lethal (SS) and wild type (NS) strains

The larval development time was similar between the wild type and the tsl strains, taking approximately 10 days from L1 to pupation (Mann-Whitney test, *P* = 0.081). For both strains, pupae were collected between day 7 and day 13 with the maximum collected on day 9 and 10 (Fig. 7). The proportion of adults emerged from pupae was similar between the two strains: 82.44% and 85.48% for the tsl and wild type strain, respectively (Fisher's exact test, *P* = 0.609).

**Fig. 8:**
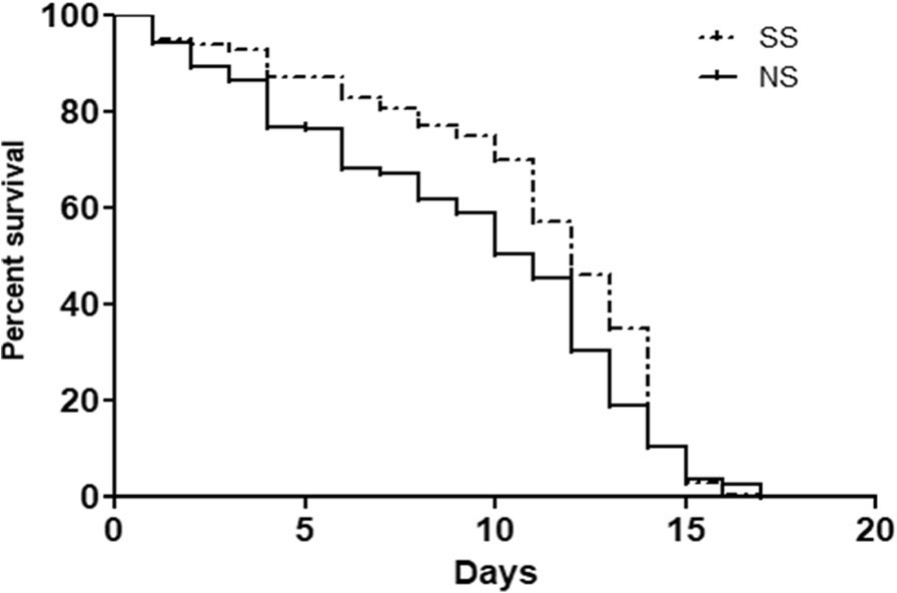
Longevity of adult mosquitoes of the temperature sensitive lethal (SS) and wild type (NS) strains

Regarding the longevity, tsl mosquitoes tended to live longer than wild type ones, with a mean survival of 12 and 11 days, respectively. The Log-rank (Mantel-Cox) test confirmed that there is statistically significant difference in the survival curves of the tsl and wild type strains (*P* < 0.001) (Fig. 8).

## Discussion

In this paper, we report the isolation of a tsl mutation, for the establishment and characterization of an *An. arabiensis* temperature-sensitive strain towards its potential use for the development of a GSS for SIT applications against this malaria vector [16]. This species belongs to the *An. gambiae* complex comprising eight species among which *An. arabiensis*, *An. coluzzii* and *An. gambiae* are the major vectors [28]. *Anopheles arabiensis* is a temporary pool breeding mosquito species prevailing in arid savanna zones and for such its populations experience dramatic reduction during the dry season. This seasonality makes *An. arabiensis* a suitable species to be targeted by SIT since releases done during dry seasons could easily allow to achieve suppression of low-density populations. Moreover, the SIT-targeted species should be genetically uniform, panmictic (i.e. freely mating) and preferably there should be only one species present in the release area, and this appears to be the case for *An. arabiensis* [31].

The *An. arabiensis* tsl strain was isolated following treatment of wild males from North Cameroon with EMS, an alkylating agent which is known to induce mutations in various organisms including *Anopheles* [30, 32–34]. Treatment of male adult mosquitoes with 0.05% EMS during 24 h did not significantly reduce female fecundity or fertility. Similar observations were reported in *Aedes* mosquitoes even after five days of exposure to EMS [35]. The fact that high sterility was observed in *An. arabiensis* after only two days of treatment with EMS in this study indicate that this species would be more sensitive to this mutagen than *Aedes* mosquitoes. This high sterility could have been caused by chromosomal breakages due to prolonged effect of EMS [36]. Our study shows that the EMS induced a tsl mutation in *An. arabiensis* and this finding is in line with EMS mutagenic effects in other organisms. For example, it has been reported that most EMS-induced recessive lethal mutations in *Drosophila* appeared to be point-mutations and that few EMS lethals were associated with chromosomal rearrangements or deletions [37].

In SIT programmes, tsl-based sexing methods allow removal of females at early developmental stage, ideally during egg stage, and avoid the use of toxic chemicals. Using EMS, several tsl mutations have been induced and used as selectable markers in insects of medical or economic importance including the vector mosquito *C. tritaeniorhynchus* and the fruit fly *C. capitata.* For *C. tritaeniorhynchus*, the tsl isolated was recessive and located on an autosome. Females homozygous for tsl survived at 26°C but died at 32°C. This tsl locus has the particularity to be located within a chromosomal inversion that suppresses recombination allowing indefinite maintenance of the strain with no culling [38]. Similarly, the tsl isolated in *C. capitata* was also recessive and autosomal, and homozygous females died when the eggs were exposed to temperatures between 31°C and 35°C for 24 hours. A white pupae mutation located close to the tsl gene allowed the monitoring of the strain to ensure its stability [39].

In this study, preliminary crossing experiments indicated that our tsl is also recessive and autosomal. However, heterozygotes tended to be slightly more temperature-sensitive than the wild type, similarly to what has been observed in *C. capitata* [39]. The lethal conditions of homozygous females of the tsl strain were different from those of the two other studies mentioned above. For instance, homozygous females were killed when exposed as L1 to 41°C for three hours. This difference could reflect different behavior of the tsl mutations or more likely difference in temperature sensitivity between eggs and larval stages, with the eggs being the most temperature-sensitive phase of the life-cycle. Additional work will be done to characterize the sensitive period during the life-cycle by shifting mosquitoes from the permissive to the restrictive temperature and *vice versa* at different successive intervals after the eggs are laid. In addition, the temperature-sensitive profile of the other developmental stages (e.g. adults) will be investigated.

The established *An. arabiensis* tsl strain has been maintained at 25 ± 1°C and 70% relative humidity. Our data showed that this strain tends to lay less eggs than the wild type strain, but its larvae develop relatively faster and adults live longer. Before this tsl strain is used for the development of a genetic sexing strain, a thorough genetic analysis needs to be done to confirm our initial observations that the tsl phenotype is due to an autosomal recessive gene as well as to confirm the stability of the marker in different backgrounds. This is crucial because changes in the expression of temperature-sensitive lethals in outcrossing experiments with *Drosophila spp*. have been reported [40]. Besides, it was noted that many EMS-induced temperature-sensitive mutations in *Habrobracon* either disappeared or became non-temperature-sensitive when the genetic background was altered [37]. Moreover, the temperature sensitivity profile of all stages (eggs, larvae, pupae and adults) should be defined in order to determine optimal conditions for rearing and colony maintenance, particularly under mass rearing conditions and for the production of male only colonies, if a GSS is successfully developed. Finally, isolation of a traceable selectable marker linked to the *tsl* will allow for tracking of recombinants in mass-rearing systems.

## Conclusion

The successful establishment of the *An. arabiensis tsl* strain is a valuable tool towards the development of a GSS for SIT applications against this species. This will be done by an irradiation-induced translocation which would link wild type tsl allele (resistant) to Y chromosome. Males carrying this translocation should then mate with females homozygous for the tsl mutation and this would result to the establishment of the GSS in which females will be homozygous for the recessive temperature-sensitive lethal and would die when they are exposed to elevated temperatures, while males will be heterozygous and would survive. Such a development would be extremely useful for the implementation of an SIT program to suppress populations of *An. arabiensis*, which is a major African malaria vector.

## Abbreviations

EMS: Ethyl methanesulfonate
GSS: genetic sexing strain
IAEA: International Atomic Energy Agency
IPCL: Insect Pest Control Laboratory
L1: First-instar larvae
OCEAC: Organisation de Coordination pour la lutte contre les Endémies en Afrique Centrale
RH: relative humidity
SIT: sterile insect technique
tsl: temperature-sensitive lethal
